# Decreased Expression of *Arginine-Phenylalanine-Amide-Related Peptide-3* Gene in Dorsomedial Hypothalamic Nucleus of Constant Light Exposure Model of Polycystic Ovarian Syndrome

**DOI:** 10.22074/ijfs.2018.5206

**Published:** 2018-01-15

**Authors:** Zahra Shaaban, Mohammad Reza Jafarzadeh Shirazi, Mohammad Hossein Nooranizadeh, Amin Tamadon, Farhad Rahmanifar, Somayeh Ahmadloo, Amin Ramezani, Mohammad Javad Zamiri, Iman Razeghian Jahromi, Fatemeh Sabet Sarvestani, Omid Koohi Hosseinabadi

**Affiliations:** 1 Department of Animal Sciences, College of Agriculture, Shiraz University, Shiraz, Iran; 2 Stem Cell Technology Research Center, Shiraz University of Medical Sciences, Shiraz, Iran; 3 Department of Basic Sciences, School of Veterinary Medicine, Shiraz University, Shiraz, Iran; 4 Department of Medical Biotechnology, School of Advanced Medical Sciences and Technology, Shiraz University of Medical Sciences, Shiraz, Iran; 5 Institute for Cancer Research, Shiraz University of Medical Sciences, Shiraz, Iran; 6 Cardiovascular Research Center, Shiraz University of Medical Sciences, Shiraz, Iran; 7 Comparative and Experimental Medicine Center, Shiraz University of Medical Sciences, Shiraz, Iran

**Keywords:** Constant Light, Dorsomedial Hypothalamic Nucleus, Polycystic Ovary Syndrome, Rats, RFamide-Relat- ed Peptide-3

## Abstract

**Background:**

An abnormality in pulse amplitude and frequency of gonadotropin releasing hormone (GnRH) secretion is the most characteristics of polycystic ovarian syndrome (PCOS). On the other hand, arginine-phenylalanine-amide (RFamide)-related peptide-3 (RFRP3) inhibits the secretion of GnRH in mammalian hypothalamus. The current study performed in order to investigate the expression of *RFRP3* mRNA in the dorsomedial hypothalamic nucleus (DMH) after the induction of PCOS in a rat model of constant light exposure, and the possible role of parity on occurrence of PCOS.

**Materials and Methods:**

In the experimental study, female nulliparous (n=12) and primiparous (n=12) rats were randomly subdivided into control and PCOS subgroups (n=6). PCOS were induced by 90 days exposure to constant light. After 90 days, blood, brain, and ovaries were sampled. Serum levels of follicle stimulating hormone (FSH), luteinizing hormone (LH), and testosterone were evaluated. In addition, six adult female ovariectomized rats as a control of real-time polymerase chain reaction (PCR) tests were prepared and in the DMH of all rats, the relative mRNA expression of *RFRP3* was assessed.

**Results:**

Histological evaluation of ovaries represented the polycystic features. In addition, serum concentrations of testosterone in the PCOS subgroups were more than the controls (P<0.05). Furthermore, the relative expression of *RFRP3* mRNA in PCOS subgroups was lower than the controls (P<0.05).

**Conclusion:**

Constant light model of the PCOS-induced rats decreased the gene expression of *RFRP3* in the DMH that suggests the decrease of RFRP3 may reduce its inhibitory effect on GnRH during the PCOS pathogenesis. This effect was stronger in the nulliparous rats than the primiparous.

## Introduction

Polycystic ovarian syndrome (PCOS) as an complex endocrine disorder in women is accompanied with ovarian dysfunction, metabolic disorders (e.g., obesity), and a myriad of causes, including genetic abnormalities, fetal epigenetic alterations, maternal or postpubertal hormonal imbalances, lifestyle, and environmental factors have been explained (1), have been proposed to explain PCOS. However, and despite the prevalence of PCOS and its effects on health, the causes of this syndrome, especially in hypothalamus, have been not completely understood.

Common neuroendocrine disorder of PCOS, increased frequency and amplitude pulses of gonadotropin releasing hormone (GnRH) (2). Furthermore, elevated secretion and pulse amplitude and frequency of luteinizing hormone (LH) release are the prominent pathophysiological features of PCOS (2, 3). LH secretion increment in 70% of women with PCOS has been accessed; this increase accompanied with increases in LH/follicle stimulating hormone (FSH) ratio (3). Suppression of FSH secretion inhibits proper development of follicles and overproduction of LH led androgen synthesis in follicular theca cells (2). Therefore, measuring of FSH and LH levels in PCOS will reflect a GnRH pulse frequency. Increase of both amount and pulse frequency of GnRH have importance in pathophysiology of PCOS.

On the other hand, some evidences show that PCOS may be originated from dysfunctions in regulating neuronal circuits of negative feedback of steroids to hypothalamus- pituitary-gonads (HPG) axis (4). Furthermore, it is possible that change in GnRH release inhibitors, such as arginine-phenylalanine-amide (RFamide)-related peptide- 3 (RFRP3), may control the hormonal irregularities of PCOS. RFRP3 neurons localize in the dorsomedial hypothalamic nucleus (DMH) of rat brain (5). Furthermore, peripheral or intracerebroventricular injection of RFRP3 inhibited LH secretion in sheep (6). Although, there is evidence supporting the existence of the *RFRP3* receptor in the pituitary, but the inhibitory effect of RFRP3 neurons on GnRH at the level of hypothalamus were achieved (7). In general, the inhibitory signals of RFRP3 on GnRH neurons allow preovulatory LH surge happen at the right time (8). Also, there are reports that show PCOS is common in nulliparity and multiple gravidity can reduce PCOS (9). In the present study a constant light model was induced in both nulliparous and uniparous rats to evaluate the mRNA expression of RFRP3 in the DMH of PCOS rats.

## Materials and Methods

All experimental procedures on rats were performed based on the instructions of Animal Care Committee of Shiraz University. The experimental procedure had been approved by Chancellor of Research Committee of the Shiraz University.

### Animal and polycystic ovarian syndrome induction

In the experimental study, 30 female Sprague-Dawley rats were purchased from and kept in the Center of Comparative and Experimental Medicine, Shiraz University of Medical Sciences. The rats were housed in standard condition of laboratory animal center (22 ± 1°C temperature) and food and water were available ad libitum. Twelve nulliparous (38 days-old, 177 ± 20 g) and 12 uniparous (80 days-old, 226 ± 20 g) rats were randomly allocated into two PCOS and control normal sub-groups (n=6). PCOS was induced using constant light (10). Briefly, the both the PCOS sub-groups were exposed to 90 days constant 24 hours per day fluorescent light with 350 lux intensity to 1 m^2^ on floor. The control normal sub-groups were housed in 12 hours light to 12 hours dark condition. After 90 days, blood, brain and ovaries of the PCOS and control rats were collected.

Six remained nulliparous rats were used as the ovariectomized control group for real-time polymerase chain reaction (PCR). The ovariectomy was performed through a ventral midline incision after anesthetizing with an intraperitoneal injection of xylazine (7 mg/kg, Alfazyne, Netherlands) and ketamine (100 mg/ kg, Netherlands). Brain tissue sampling in this group was done after two weeks.

### Sampling and histological evaluation

For sampling, the rats were euthanized with ether and blood was sampled in tubes without anticoagulants by cardiac puncture. Serum was collected by centrifuging 2000 rpm for 15 minutes and then stored at -80°C until analysis.

Brain was dissected out from skull and DMH was sampled (11). In brief, the diencephalon was rapidly separated in cold condition by an anterior coronal section to the optic chiasm and a posterior coronal cut at the mammillary bodies. To separate DMH from anteroventral periventricular nucleus, the third coronal sectioning was performed through the middle of the optic tract and rostral to infundibulum. The samples were covered in aluminum foil and rapidly stored in liquid nitrogen.

Then ovaries were removed through ventral midline incision and kept in 10% buffer formalin solution. Ovaries were dehydrated by graded concentrations ethanol and xylene and then were embedded in paraffin. Serial sectioning was performed at thicknesses of 10 µm. Sections were deparaffinized in 60°C. In room temperature, sections were rehydrated in xylene and graded concentrations of ethanol. Samples were stained with hematoxylin and eosin (H&E) stain. Follicle types in ovarian sections were defined (12) with light microscope (CX21, Olympus, Japan).

### Measurements of serum hormone 

Serum concentration of testosterone with 0.2 nmol/L sensitivity (catalog# RK-61M, Institut des Isotopes Ltd, Hungary) was measured by radioimmunoassay (RIA) technique. In addition, serum concentrations of follicle stimulating hormone with 0.09 mIU/mL sensitivity (catalog# RF01N, Gyeonggi-do, South Korea) and luteinizing hormone with 0.22 mIU/mL sensitivity (catalog# RF03N, Gyeonggi-do, South Korea) were determined using immunoradiometric assay (IRMA) technique.

### *RFRP3* expression by real-time polymerase chain reaction

*RFRP3* mRNA relative expression in DMH of rat brains was measured (13). Total RNA from DMH was extracted from the frozen brain samples after grounding it in liquid nitrogen and adding extraction buffer by Tripure isolation reagent kit (Roche Life Science, Branford, CT) according to manufacturer's instructions. Brain samples were transferred to the free RNase microtube and after mixing the solution were kept at room temperature for 5 minutes. Then, 0.2 ml chloroform was added to the solution and was held at room temperature for 15 minutes. Afterwards, the supernatant phase of mixture was transferred to another microtube after centrifuging of mixture at 12,000 rpm for 20 minutes. The same volume of isopropanol was added to the microtube. After washing the RNA pellet with 75% ethanol, it was quickly dried. The total purified RNA was measured by spectrophotometer (Nano-Drop ND-1000, Nano-Drop Technologies, Wilmington, DE, USA). To ensure the removal of genomic contamination, the DNase treatment was done using a DNase kit (Fermentas, St. Leon-Roth, Germany). The first strand cDNA synthesis using cDNA synthetize kit was performed (Fermentas, St. Leon-Roth, Germany). Primers for *RFRP3* target gene and rat ß-actin reference gene were designed using Allele ID software version 6.0 (Premier Biosoft International, USA). Relative real-time PCR reactions was performed by 20 µL real time master mix (Yekta Tajhiz Azma, Iran) containing 1 µL cDNA, 4 pmol of primer, and 1X SYBR Green buffer. The cDNA samples were amplified (Table 1) by a StepOne cycler (Applied Biosystems, CA, USA). Amplification condition was 15 minutes at 94°C, 40 cycles of 94°C 10 seconds, 58°C 15 seconds, and 72°C 30 seconds for RFRP and 15 minutes at 94°C, 40 cycles of 94°C 15 seconds, 57.8°C 20 seconds, and 72°C 30 seconds for ß-actin.

**Table 1:** Designed primers for *arginine phenyl alanine related peptide-3 (RFRP3)* and *β-actin* genes and their amplification reaction conditions


Gene	Primer sequencing (5ˊ-3ˊ)	Amplicon length (bp)

*RFRP3*	F: AAGACACTGGCTGGTTTG	192
	R: TTGAAGGACTGGCTGGAG	
*β-actin*	F: CCACACTTTCTACAATGAGC	169
	R: ATACAGGGACAACACAGC	


For quantitative assessment and evaluation of the relative mRNA expression of *RFRP3* gene the CT values were estimated with real-time PCR Step One software version 2.1 (Applied Biosystems, CA, USA). Accordingly, CT of *RFRP3* and CT of reference gene were entered in the 2^-ΔΔCT^ equation. ΔCT is a difference between the internal control gene CT value and the target gene CT value. ΔΔCT was obtained by subtracting the ΔCT of each sample from the average of CT value of calibrators (ovariectomized rats).

### Statistical analysis

The normality of data from hormone measurements and the *RFRP3* mRNA relative expression were evaluated by the Kolmogorov-Smirnov test. Then, they were analyzed by one-way ANOVA (SPSS for Windows, version 20, SPSS Inc, Illinois), and mean differences was compared by post hoc LSD test at P<0.05. Data are presented as mean and SE.

## Results

### Histological evaluation of ovaries

**Fig.1:**
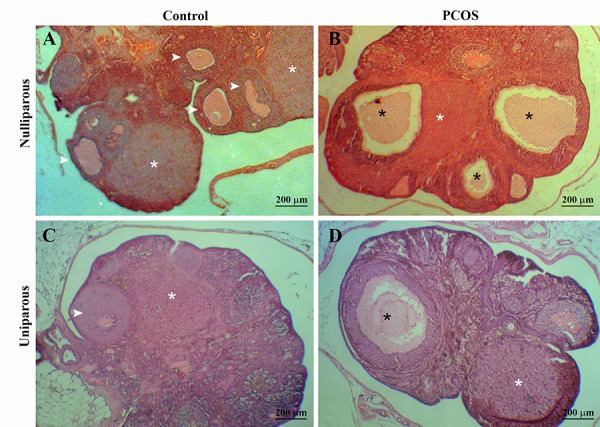
Alterations of histological charecters of ovaries in the female nulliparous and the primiparous rats after the exposition to continuous light during 90 days. The control groups show normal ovarian feature with Several corpus luteum (white stars) and normal tertiarry follicles (arrows). The polycystic ovary syndrome (PCOS)-induced groups showed considerably distended and cystic tertiary follicles [black stars, hematoxilin and eosin staining (H&E)]. A. Nulliparous control, B. Nulliparous PCOS, C. Uniparous control, and D. Uniparous PCOS.

**Fig.2:**
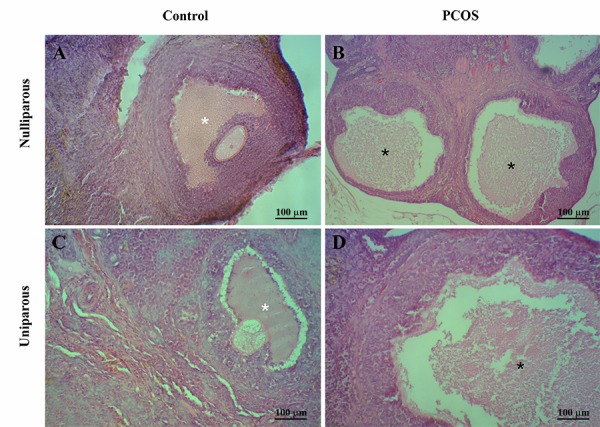
Alterations of tertiarry follicles features in the female nulliparous and the primiparous rats after continuous light exposure during 90 days. Ovary of the control rat with normal tertiary follicles (white stars). Oocytes and corona radiata are absent in the polycystic ovary syndrome (PCOS)-induced groups and atretic follicles (black stars) are more observable [hematoxilin and eosin staining (H&E)]. A. Nulliparous control, B. Nulliparous PCOS, C. Uniparous control, and D. Uniparous PCOS. [hematoxilin and eosin staining (H&E)]. PCOS; Polycystic ovary syndrome.

**Fig.3:**
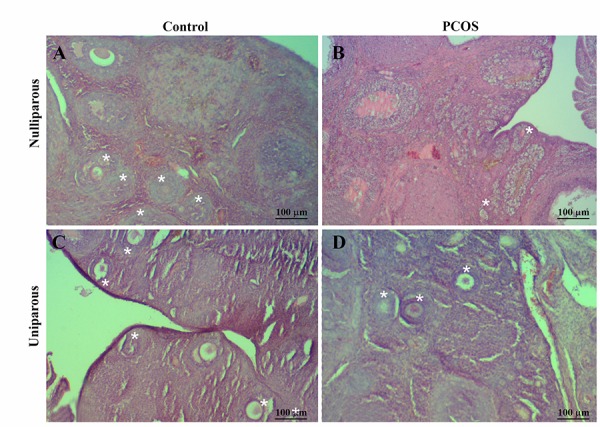
Decrease in the number of secondary follicles (stars) in ovary of the rat model of polycystic ovary syndrome (PCOS) in comparison with the control rat [hematoxilin and eosin staining (H&E)]. A. Nulliparous control, B. Nulliparous PCOS, C. Uniparous control, and D. Uniparous PCOS. [hematoxilin and eosin staining (H&E)]. PCOS; Polycystic ovary syndrome.

**Fig.4:**
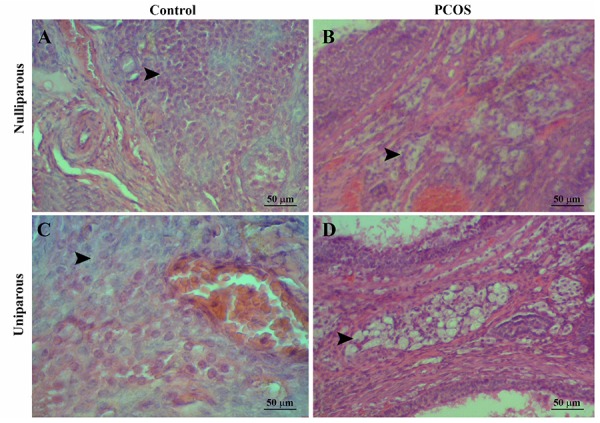
Hypertrophied and hyper-vaculated stromal cells in the ovarian medula of the polycystic ovary syndrome (PCOS) rats in comparison with normal stromal cells in the control rats [hematoxilin and eosin staining (H&E)]. A. Nulliparous control, B. Nulliparous PCOS, C. Uniparous control, and D. Uniparous PCOS. [hematoxilin and eosin staining (H&E)]. PCOS; Polycystic ovary syndrome.

The microscopic evaluation of ovaries in the PCOS groups showed formation of cystic follicles (Fig .1B, D) in comparison with controls (Fig .1A, C) and alterations of thickness and structure of follicular wall layers (Fig .2B, D) in comparison with controls (Fig. 2A, C). Active corpora lutea was not observed in the PCOS rats, but in the control group numerous corpora lutea were obvious (Fig .1). The number of the secondary follicles was lower in the PCOS rats compared with control rats (Fig .3). In medulla, stroma cells’ cytoplasm of the PCOS rats demonstrated high amount of vesicles in comparison with controls (Fig .4).

**Fig.5:**
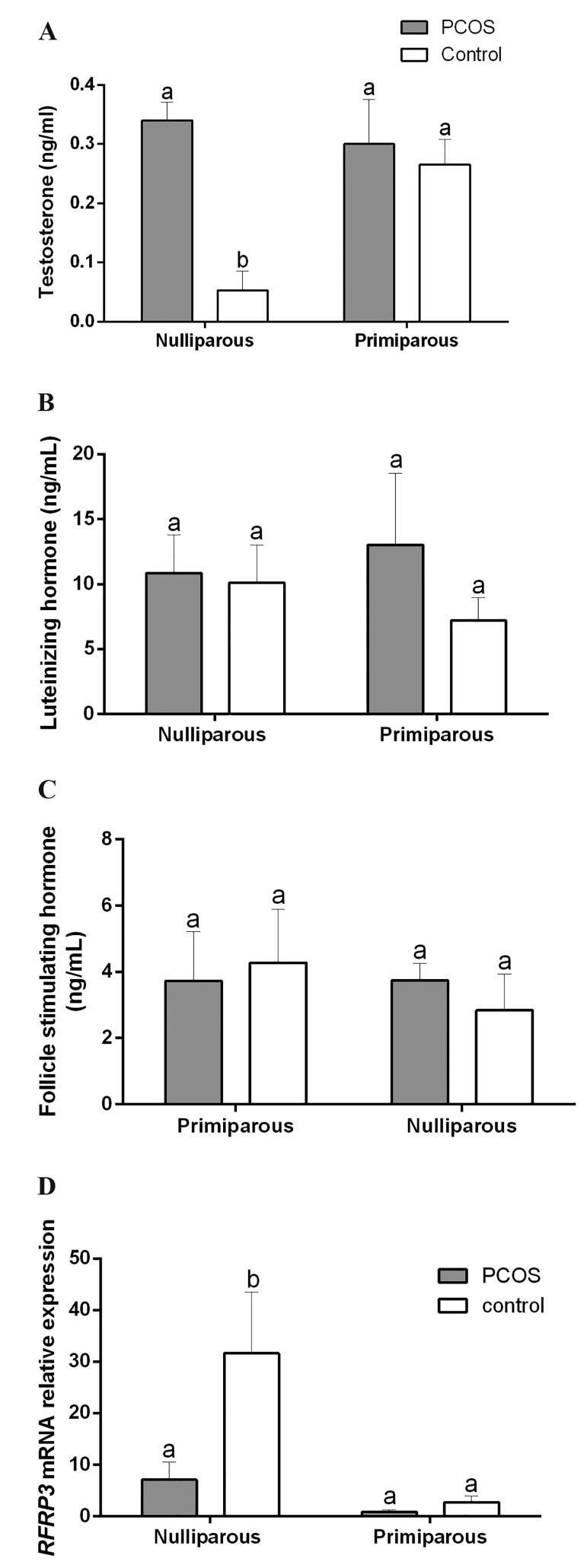
Alterations of the mean (+SE) of serum hormone concentrations in the female nulliparous and the primiparous rats after the exposition to continuous light during 90 days for induction of polycystic ovary syndrome (PCOS). A. Testestrone, B. Luteinizing hormone (LH), C. Follicle stimulating hormone (FSH), and D. Decrease in the mean (+SE) expression of *arginine phenyl alanine related peptide-3 (RFRP3)* mRNA in the dorsomedial nucleus of hypothalamus of nulliparous PCOS-induced. a,b; Different letters show statistically significant differences between groups (P<0.05).

Serum testosterone concentrations of nulliparous the PCOS rats was more than the nulliparous control (Fig .5A, P<0.05), but not significantly different from the uniparous rats (P>0.05). FSH and LH concentrations were not significantly different between the control and the PCOS sub-groups (Fig .5B, C, P>0.05).

### The *RFRP3* gene expression in hypothalamus

The real-time PCR analysis showed that the expression of *RFRP3* gene in the PCOS groups reduced (Fig .5D, P<0.05). Expressions of *RFRP3* gene in the uniparous rats of the PCOS and the control sub-groups were not different (P>0.05).

## Discussion

The present study for the first time showed that the PCOS induction by constant light decreases *RFRP3* gene expression in the DMH of rats in the nulliparous group, an effect that was not observed in the uniparous group. Consistent with our finding, in a prepubertal rat model of androgen-induced PCOS, reduction in the transcription of RFRP3 inhibitory neuropeptide in whole hypothalamus has been recently reported (14). Furthermore, in letrozole-induced PCOS rats, gene expression of *RFRP3* and increased in *RFRP3* receptor in pituitary was observed. Although, exogenous hormones may alter the pathogenesis of PCOS in those models, our findings, similar to (15), shows the role of RFRP signaling in PCOS.

In women suffering from PCOS, the concentrations of LH increases and FSH decreases in comparison with healthy women (16). Interestingly, in the current study, long term constant light (90 days) with intensity of 350 lux increased the mean concentrations of LH and decreased the mean of concentrations FSH in the uniparous rats in comparison with the control group but the alterations were not significant. Consistent with our findings, 100 days constant light exposures with about two times illumination intensity (500-600 lux) in rats could induce higher LH and lower FSH concentrations than the control group (17). While, long term exposure to continuous luminescence much lower than 350 lux (the rats were kept in a room with light) could slightly increase LH hypersecretion in rats (18), short-term continuous exposure to light (3 days) could suppress the synthesis of LH in female rat by reducing the sensitivity of the LH-releasing hormone release centers to estrogen (19). Therefore, increasing the intensity of the light could induce alterations, akin to those observed in PCOS rat models, to in gonadotropins concentrations in human PCOS.

Hyperandrogenism is accepted as an important attribute of PCOS; therefore, in most animal models of PCOS, androgen hormones have been used to stimulate the PCOS (20, 21). Although, these androgen models or other hormone-induced models of PCOS, especially in the prepubertal and pubertal models, have been used for evaluation of hypothalamic functions in PCOS, but there is a concern that these exogenous hormones may directly disturb the neuronal circuits and the observed alternations are not related to the induced PCOS. Therefore, prenatal androgen models or non-hormonal induced models of PCOS, such as constant light model of PCOS (10), may demonstrate closer hypothalamic features of PCOS to the human PCOS than the others.

Increase in serum concentrations of testosterone in the PCOS rats of the nulliparous group compared to the controls showed the efficiency of this model for evaluation of PCOS in hypothalamus without exogenous androgens. Consistent with our findings, increase in serum testosterone levels of rats that were exposed to 112 days constant light exposures with illumination intensity of 600 lux was shown (22). 

To explain this phenomenon, it has been shown that the testosterone concentrations are positively correlated with the expression of the androgen receptor in the hypothalamic suprachiasmatic nucleus (SCN). This locus regulates circadian rhythms and light exposure controls it (23). On the other hand, SCN sends direct and indirect projections to DMH (24), which suggests the role of SCN in control of DMH reproductive function, and can also explain the observed relationship between testosterone increase and constant light exposure. Furthermore, it has been shown that SCN projects to RFRP neurons in DMH of hamster (25). Therefore, our findings in combination with previous findings, suggest the relationship of RFRP3 in DMH and testosterone effects on SCN. However, clarifying this pathway in pathogenesis of PCOS needs further investigation.

Histopathologic evaluation of ovaries showed that continuous light exposure increased the number of antral follicles and atretic follicles. Consistent with our results, increase in large antral and atretic follicles and reduction of the number of early growing follicles have been previously reported in rats that were subjected to 13 weeks of continuous exposure (26). Continuous light exposures of rats for 100 days led to atresia of ovarian follicles due to lack of preovulatory LH surge and resulted in cyst formation (17). Although, corpora lutea were present in the uniparous PCOS-induced group, but the absence of corpus luteum is another attribute of PCOS in the current study in the nulliparous rats, in accordance with a previous report (26).

However, the nulliparous group represented a better PCOS model than the primiparous rats, but it is not clear if gravidity can influence the occurrence of PCOS or not. Consistent with the current finding, in the consistent light model of PCOS, the PCOS criteria in the nulliparous rats were more than the primiparous group. In women, obesity would exacerbate the insulin resistance, a predisposing factor for PCOS. It has been reported that pregnancy can be a risk factor for obesity (27). Therefore, it can be expected prevalence of PCOS increase in the uniparous women. While, evidences showed that nulliparous women are susceptible to PCOS (28). Therefore, based on the current findings and the other published reports it can indirectly conclude that increase in gravidity may be associated with decreased PCOS, although confirmation of it and knowing its mechanism need further investigations.

## Conclusion

The constant light model of PCOS induced decrease in the gene expression of *RFRP3* in the DMH that suggests the decrease in RFRP3 reduces its inhibitory effect on GnRH in the PCOS pathogenesis. The Continuous light exposure model of PCOS in rats could trigger the creation of phenotypic traits of PCOS with similar histopathologic and hormonal phenomenon in human PCOS. Furthermore, by removing exogenous androgen, this model can be applied to hypothalamic-pituitary-ovarian disorders in PCOS studies.
